# The Role of Metformin in the Management of NAFLD

**DOI:** 10.1155/2012/716404

**Published:** 2011-12-12

**Authors:** Angela Mazza, Barbara Fruci, Giorgia Anna Garinis, Stefania Giuliano, Roberta Malaguarnera, Antonino Belfiore

**Affiliations:** Endocrinology Unit, Department of Clinical and Experimental Medicine, University Magna Graecia of Catanzaro, Campus Universitario, Località Germaneto, Viale Europa, 88100 Catanzaro, Italy

## Abstract

Nonalcoholic fatty liver disease (NAFLD) is the most common liver disorder worldwide. Its prevalence ranges 10–24% in the general population, reaching 60–95% and 28–55% in obese and diabetic patients, respectively. Although the etiology of NAFLD is still unclear, several lines of evidences have indicated a pathogenetic role of insulin resistance in this disorder. This concept has stimulated several clinical studies where antidiabetic drugs, such as insulin sensitizers including metformin, have been evaluated in insulin-resistant, NAFLD patients. These studies indicate that metformin might be of benefit in the treatment of NAFLD, also in nondiabetic patients, when associated to hypocaloric diet and weight control. However, the heterogeneity of these studies still prevents us from reaching firm conclusions about treatment guidelines. Moreover, metformin could have beneficial tissue-specific effects in NAFLD patients irrespective of its effects as insulin sensitizer.

## 1. Introduction

Nonalcoholic fatty liver disease (NAFLD) is the most common cause of chronic liver disease. It includes a broad spectrum of liver alterations, ranging from pure steatosis to cirrhosis, through nonalcoholic steatohepatitis (NASH). NAFLD is characterized by liver damage and functional impairment similar to those observed in alcoholic liver disease although occurring in patients who do not drink or drink only a moderate amount of alcohol [1].

Although the pathogenesis of the disorder is not fully clarified, insulin resistance is widely considered a pivotal feature of NAFLD, which is strongly and independently associated with increased risk of type 2 diabetes mellitus (T2DM) and cardiovascular disease [2]. In the past, the diagnosis of NAFLD was mostly the result of ultrasound investigations or liver function tests performed at random. Nowadays, the great attention paid to the occurrence of metabolic syndrome and its related aspects has allowed us to put more emphasis on diagnosis and treatment of this disorder. Clinicians have now become to pay attention to early diagnosis and treatment of NAFLD in insulin-resistant obese and T2DM patients. Diet and lifestyle changes are a mainstay in the management of these patients, while specific pharmacologic treatment for NAFLD is so far lacking. Therefore, it is not surprising that several studies have evaluated the efficacy of insulin sensitizers in NAFLD patients. Among insulin sensitizers, metformin has recently acquired a central role in the treatment of T2DM and other disorders associated with insulin resistance, such as polycystic ovary syndrome (PCOS), and both experimental and clinical studies have recently supported the use of metformin as a useful adjunct in NAFLD patients.

We will review evidences concerning a possible use of metformin in the treatment of NAFLD.

## 2. Epidemiology and Risk Factors

Epidemiological studies on NAFLD are limited by the lack of a universal screening method used for diagnosis and by the presence of various definitions and diagnostic criteria. However, NAFLD has been reported to occur in 10–24% of the general population [3, 4], thus, representing the most common cause of elevated liver enzymes and one of the most common forms of liver disease in the world. The occurrence of NAFLD is increasing not only in Western countries, but also in Eastern countries, where evidence of steatosis at liver ultrasounds (USs) is found in 16–30% of the general population [1]. Moreover, NAFLD is increasingly diagnosed in children and adolescents together with the concomitant increase in obesity. Small epidemiological studies have found that NAFLD occurs in 2.6–25% of obese children [5].

As already mentioned, NAFLD is strongly associated with insulin resistance and other components of the metabolic syndrome, like T2DM, central obesity, hyperlipidemia, hypertension, and with other conditions associated to insulin resistance, such as hyperuricemia, atherosclerosis, and PCOS [6, 7]. For this reason, NAFLD is now regarded as the hepatic manifestation of the metabolic syndrome, affecting up to a third of the general population [3, 4]. In fact, the occurrence of NAFLD is reported to range 28–55% in T2DM patients, 60–95% in obese patients, and 27–92% in hyperlipidemic patients [8]. Moreover, the strongest association of NAFLD is with central adiposity, which is an important risk factor even in patients with a normal body mass index (BMI). Diet, lack of exercise, and possibly small bowel bacterial overgrowth are candidate factors influencing the risk of NAFLD [3]. Finally, several studies have reported a potential role of genetic factors for the development of NAFLD. However, these studies have limitations and must be interpreted with caution [3].

## 3. Pathogenesis

The pathogenesis of NAFLD is still object of discussion. The prevailing hypothesis is based on the “two hits” model, proposed by Day and James in [9]. The “first hit,” characterized by free fatty acid and triglyceride accumulation in liver (steatosis), is caused by insulin resistance (through lypolysis and hyperinsulinemia) and obesity (through leptin resistance). This induces a chronic inflammatory condition characterized by the release of proinflammatory cytokines and by oxidative stress, both of which are responsible of the “second hit,” which induces the progression from steatosis to more advanced stages of liver damage (steatohepatitis and fibrosis). The “two hit” model has been recently challenged because an increased ratio of saturated-to-unsaturated fatty acids delivered to or stored within the liver may, in part, mediate the progression from simple steatosis to NASH. Proof of this concept has been provided by recent data showing that when triacylglycerol (TAG) precursors accumulate in the liver and the mechanisms of hepatic detoxification are overwhelmed or inactive, saturated fatty acids directly induce hepatic inflammation and insulin resistance both of which may result in steatosis progression toward more severe stages of liver disease [10]. Although the molecular mechanisms that mediate the effects of saturated fatty acids are still unclear, it has been suggested that free fatty acids and TAG metabolites (fatty acyl-CoA, diacylglycerol, ceramide) directly or via Toll-like receptors 2 and 4 (TRL2 and 4) induce endoplasmatic reticulum stress, mitochondrial disfunction, ROS production, impaired hepatic protein metabolism, inhibition of insulin signaling, and activation of inflammatory pathways (NF-*κ*B, JNK, IKK) [11]. Moreover, as suggested by novel evidences, fatty liver releases in the circulation factors called hepatokines (i.e., fetuin A, sex hormone-binding globulin (SHBG), and selenoprotein P) that are directly involved in the pathogenesis of local and system inflammation and in peripheral and hepatic insulin resistance [12]. However, it is important to emphasize that insulin resistance remains a key player in NAFLD pathogenesis and that fatty liver may, in turn, potentiate insulin resistance. It remains controversial whether NAFLD may contribute to insulin resistance independently of the effect of age and total adiposity [13]. Studies showing that liver fat content affects insulin sensitivity in humans more strongly than visceral fat [14, 15] support a direct and important role of fatty liver in the pathogenesis of insulin resistance.

## 4. Diagnosis

NAFLD is essentially an asymptomatic condition. In patients with NAFLD, who do not have advanced liver disease, the most common sign on physical examination is hepatomegaly, and the diagnosis is made when a US or radiological test reveals evidence of fatty liver. Mild to moderated elevation of ALT, AST, or both are the most common findings (with the AST: ALT ratio <1) [16]. However, liver enzymes may be normal in up to 78% of patients, and; thus, enzyme elevation is insensitive for the detection of NAFLD [17].

The diagnosis of NAFLD is made after exclusion of other causes of liver disease, such as alcohol abuse, viral hepatitis, autoimmune disorders. Older age, obesity, T2DM are risk factors suggesting potential NAFLD diagnosis. Abdominal US is currently the most common method employed for qualitative assessment of hepatic steatosis, because it is noninvasive and widely available.

Both computerized tomographic (CT) scanning and, in particular, magnetic nuclear resonance (MNR) imaging seem to be more sensitive techniques for the quantification of liver steatosis. However, none of these imaging techniques have sufficient sensitivity and specificity for staging the disease, and they cannot distinguish between simple steatosis and fibrosis [18]. Liver biopsy still remains the gold standard for distinguishing between the broad range of chronic liver diseases, but it is limited by its cost, the potential risk of bleeding, and the absence of consensus regarding the histopathological criteria that firmly define NASH and differentiate between NAFLD entities [19].

Given the high prevalence of NAFLD, it would be desirable to use in clinical practice a more practicable and noninvasive diagnostic method. In a recent study, US showed high sensitivity (91.7%) and specificity (100%) in detecting fatty liver [20], and a recent review confirmed that US can accurately identify steatosis with a sensitivity and a specificity of 80–100% [19]. Moreover, the accuracy of US in detecting steatosis seems to be unaffected by obesity [21]. Finally, a recent prospective study [22] demonstrated that serial liver US is an accurate tool for noninvasive monitoring of efficacy of interventions in NAFLD patients.

### 4.1. Emerging Diagnostic Tools

There is mounting evidence that cytokines secreted not only from adipose tissue, namely, adipokines, but also from hepatocytes in response to liver injury, are involved in the pathogenesis of NAFLD as well as in its progression [23]. Clinical studies suggest that serum levels of leptin, resistin, adiponectin, tumor necrosis factor *α* (TNF *α*), interleukin-6 (IL-6), visfatin, CK-18, and retinol binding protein 4 (RBP4) differ among patients with NAFLD and NASH and healthy controls [24–26].

To overcome biopsy limitations, noninvasive methods have been developed and validated for differentiating simple fatty liver from NASH and for predicting the risk of NAFLD evolution to NASH. These alternative surrogate markers of liver fibrosis include liver stiffness measurements (LSMs), using the method of transient elastography (Fibroscan) [27] and various algorithms, among which the NAFLD fibrosis score [28], the BAAT score [29], and the HAIR score [30], all based on biochemical and clinical parameters. These algorithms have all been developed as simple noninvasive scoring systems aimed at separating patients with NAFLD with and without advanced liver fibrosis by using routinely determined and easily available clinical and biochemical variables [28].

However, currently, the debate is also open on this issue, and new studies are needed.

## 5. Strategies in NAFLD Management

The first-line treatment of NAFLD is currently based on diet and lifestyle modifications. Most of the published studies in NAFLD population have shown that gradual weight loss (5–10%), calorie-restricted diet, and regular physical exercise lead to a decrease in the incidence of metabolic syndrome, improvement in liver enzyme profile, and resolution of hepatic steatosis [31–34]. However, most of these studies are nonrandomized and short term. Therefore, the paucity of data has limited the production of diet and exercise evidence-based guidelines for NAFLD patients. Moreover, dietary treatment is limited by the lack of compliance and the frequent regain of weight at followup [35].

A pharmacological treatment in patients with NAFLD is not universally accepted yet. Given that insulin resistance plays a key role in the pathogenesis of NAFLD, many studies have evaluated the use of insulin sensitizers as a possible treatment for this disease. Biguanides (metformin) and thiazolidinediones (TZDs), including pioglitazone and rosiglitazone, are the two classes of insulin sensitizers studied in humans [23].

Several trials have shown a beneficial effect of TZDs in patients affected by NAFLD. Three studies, two open-label and one placebo-controlled trial, have evaluated the efficacy of rosiglitazone in NAFLD patients [36–38]. All of these studies have reported an improvement in transaminases levels and hepatic inflammation. However, nowadays rosiglitazone has been removed from the market because of its significant side effects.

The second thiazolidinedione available for the time being is pioglitazone. Four small trials and a large-controlled trial have been conducted to evaluate its efficacy in the treatment of NAFLD [38–42]. In the largest controlled trial, Belfort et al. [40] compared diet plus pioglitazone to diet plus placebo in 55 patients. The pioglitazone-treated group showed an improvement in ALT (by 50%), steatosis (by 54%), insulin sensitivity (by 48%), liver inflammation, and ballooning necrosis but not fibrosis. An improvement in fibrosis was seen only in one of the small four studies mentioned above [41]. Significant amelioration of liver biochemistry, steatosis, and liver inflammation has been also reported in the multicenter placebo-controlled trial called PIVENS [43] (pioglitazone versus Vit E and versus placebo), in which NASH nondiabetic patients were treated for 96 weeks with pioglitazone (30 mg daily). Even if all of these studies have demonstrated the usefulness of pioglitazone in NAFLD, this drug has only been used in patients with biopsy-proven NASH. Furthermore, the long-term safety of this drug is not yet well established. For this reason, today the use of pioglitazone in some countries has been restricted for possible unknown long-term side effects [44]. Another insulin sensitizer that could be used in NAFLD patients is metformin. Its efficacy and usefulness will be extensively discussed in the next paragraph.

## 6. Role of Metformin in NAFLD

### 6.1. Mechanism of Action of Metformin

Metformin was introduced in clinical practise in the 1950s and is widely used as a first-line treatment for patients with type 2 diabetes mellitus [45]. The effectiveness of metformin as an antidiabetic drug is explained by its ability to lower blood glucose by decreasing gluconeogenesis in the liver, stimulating glucose uptake in the muscle, and increasing fatty acid oxidation in adipose tissue [46]. The final effect is an improvement of peripheral insulin sensitivity. At molecular level, some of the beneficial effects of this drug have been related to the phosphorylation and nuclear export of LKB1. This latter kinase activates adenosine monophosphate-activated protein kinase (AMPK), a regulator of energy metabolism, able to stimulate ATP-producing catabolic pathways (glycolysis, fatty acid oxidation, and mitochondrial biogenesis) and to inhibit ATP-consuming anabolic processes (gluconeogenesis, glycogen, fatty acid, and protein synthesis) [47]. Upon activation in response to energy stress, in the muscle, AMPK induces hexokinase II expression and GLUT4 gene upregulation and translocation to cell membrane, leading to an increase in glucose uptake. Furthermore, it phosphorylates and inhibits glycogen synthase, thereby, inducing a decrease in glycogen synthesis. In the liver, AMPK reduces hepatic gluconeogenesis by inducing the phosphorylation of CREB-binding protein (CBP) and, as consequence, the dissociation of the gluconeogenic CREB-CBP-TORC2 transcriptional complex [45]. This event, mediated by atypical protein kinase C (PKC *ι*/*λ*), triggers the disassembly of the transcription machinery and the inhibition of the expression of gluconeogenesis enzyme genes including phosphoenolpyruvate carboxykinase (PEPCK) and glucose-6-phosphatase (G6Pase). The activation of AMPK by metformin exerts beneficial effects also on lipid metabolism. Indeed, upon metformin-induced phosphorylation, AMPK inactivates acetyl-CoA carboxylase (ACC) and 3-hydroxy-3-methylglutaryl (HMG)-CoA reductase, decreases fatty acid synthase (FAS) expression, and activates malonyl-CoA carboxylase. The final effect is a decrease in fatty acid and cholesterol synthesis. Moreover, AMPK inhibits the sterol regulatory element-binding protein-1c (SREBP-1c), which is a transcription factor for genes involved in fatty acid synthesis [48]. SREBP-1c is induced by an excess of glucose and insulin and is inappropriately increased in NAFLD patients. Recent findings show that the effect of metformin in counteracting adipose tissue expansion occurs not only through a direct inhibition of adipogenesis but also by modulating adipokine synthesis or secretion [49]. Indeed, adiponectin, induced by metformin, directly stimulates AMPK and prevents hepatic lipid accumulation by increasing *β*-oxidation of free fatty acids and/or by decreasing their *de novo* synthesis. Furthermore, in *ob/ob* mice, a model of hepatic steatosis, it has been shown that metformin reversed hepatomegaly, hepatic fat accumulation, and ALT abnormalities, by reducing hepatic tumor necrosis factor-*α* (TNF-*α*) expression [50].

Although AMPK is the main player in mediating metformin effects, it is important to note that some metabolic actions of metformin occur in AMPK-independent manner and may be mediated by MAPK- and PKA-dependent mechanisms. As it has been shown by Zhang et al. [51], metformin exerts an inhibitory effect on catecholamine-stimulated lipolysis through decreasing cAMP production and reducing PKA and MAPK activities.

**Figure 1:**
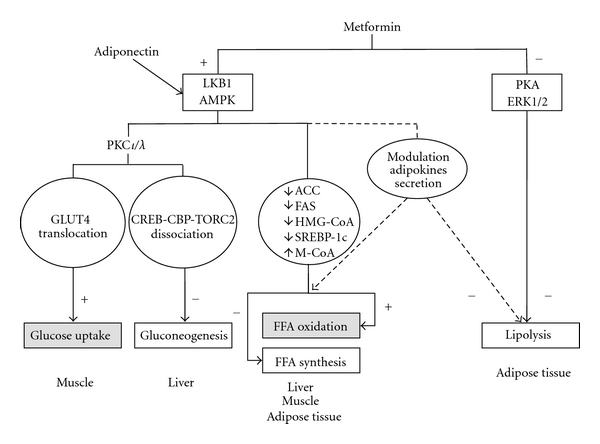
Metformin action in peripheral tissues. Metabolic effects of metformin are mainly mediated through the activation of adenosine monophosphate-activated protein kinase (AMPK), a master regulator of glucose and lipid metabolism. In skeletal muscle, metformin increases glucose uptake by enhancing the atypical protein kinase C (PKC) *ι*/*λ*-dependent glucose transporter (GLUT4) translocation to the cell membrane, while, in liver, metformin-dependent activation of PKC *ι*/*λ* reduces gluconeogenic enzyme gene expression through the dissociation of the CREB-CBP-TORC2 complex via CREB binding protein phosphorylation. In liver, muscle, and adipose tissues, AMPK decreases cholesterol and fatty acid synthesis and increases fatty acid oxidation by inhibiting the enzymes acetyl-CoA carboxylase (ACC), 3-hydroxy-3-methylglutaryl (HMG)-CoA reductase and fatty acid synthase (FAS) and activating the malonyl-CoA carboxylase (M-CoA). Moreover, it downregulates the sterol regulatory element-binding protein-1c (SREBP-1c), which is a transcription factor for lipogenetic genes. In adipose tissue, metformin inhibits lipolysis through attenuation of PKA and ERK1/2 signaling. It may also impact on the endocrine function of adipose tissue, through modulation of adipokines synthesis or secretion, probably in an AMPK-dependent manner. Adiponectin also activates AMPK, thereby, enhancing metformin action.

A schematic representation of the molecular mechanisms of metformin actions in peripheral tissues is shown in Figure 1.

### 6.2. Clinical Studies with Metformin

Several clinical trials have supported the beneficial role of metformin in patients with NAFLD (Table 1). Most of these studies have evaluated the effect of various doses of metformin on liver biochemistry (aminotransferase profile), histology, and metabolic syndrome features [35, 47, 52–59]. In 2001, Marchesini et al. [52] conducted the first pilot nonrandomized study using metformin (1.5 g day for 4 months) in 20 NASH nondiabetic patients. They observed a significant improvement in insulin resistance, aminotransferase levels, and liver morphology and volume in the treated group compared to the diet group. However, the study showed as limitation the fact that no follow-up biopsies were performed, and, thus, the histological improvement was not evaluated. Another small metformin versus diet trial, conducted in 17 randomized nondiabetic patients receiving metformin (850 mg twice a day), showed no differences in liver biopsies between treated and untreated groups. By contrast, ALT, AST, body mass index, and insulin resistance markers improved significantly in the metformin-treated group in comparison to controls [54].

Six open-label trials [47, 53, 55, 57, 58, 60] have evaluated the liver histology modification together with serum aminotransferase levels and insulin resistance markers' amelioration in NAFLD patients treated with metformin (dose ranging from 1.4 g/day to 2.0 g/day and treatment duration varying from 24 to 48 weeks) alone or in association with other drugs. All these studies reported an improvement in the indices of insulin resistance: five studies reported a reduction in liver function test values and one reported a non-significant increase of these values [53]. In terms of histological improvement, only three trials [47, 55, 57] showed significant differences in inflammation, steatosis, and fibrosis after treatment with metformin. In contrast to these promising results, some recent open-label studies have found no benefit of metformin treatment (dose ranging from 1.5 g to 1.7 g/day and for a period of 6–12 months) on liver steatosis, aminotransferase levels, and insulin resistance markers compared to lifestyle changes or control untreated group [59, 61, 62]. However, these latter studies were conducted in small series of patients. Furthermore, these controversial results could be due to the different duration and dose of the treatment and to the variable time periods between the first and the second biopsy.

Recently, we have conducted a prospective randomized study in which we evaluated the efficacy of the addiction of low dose of metformin (500 mg twice a day) to dietary treatment (1300 kcal) in 50 obese and nondiabetic patients. We found that metformin plus dietary therapy was associated with an improvement or even disappearance of hepatic steatosis similar to what observed with diet treatment alone. Metformin treatment was also associated with a significantly greater amelioration of several metabolic parameters (increased insulin sensitivity and reduced fasting glucose) than diet alone. Fasting glucose, basal serum insulin, and HOMA-IR index values decreased in both groups. However, differences both in fasting glucose (from 92.4 ± 9.9 to 89.1 ± 9.3 mg/dL, *P* = 0.04) and HOMA-IR index (from 3.3 ± 1.6 to 2.4 ± 1.2, *P* = 0.003) reached statistical significance only in the metformin group.

At baseline, impaired fasting glucose (IFG) was found in approximately 35% NAFLD patients in both groups. At the end of the study, IFG disappeared in 86% of metformin treated patients and in 62% of patients receiving only diet treatment. Metformin treatment was also significantly more effective than diet alone in reducing the proportion of patients who met the diagnostic criteria of metabolic syndrome (20% reduction in the metformin group, *P* = 0.0008 versus 4% reduction in the diet group, *P* = 0.9). Given the high proportion of NAFLD patients with metabolic syndrome and the association between metabolic syndrome and type 2 diabetes mellitus (T2DM) and cardiovascular diseases [1, 41], our results suggest that a low dose of metformin might be proposed to NAFLD patients, especially if they meet the diagnostic criteria of metabolic syndrome [35].

The potential role of metformin has also been examined in pediatric patients with NAFLD. Results in pediatric population were similar to those of adults and supported the beneficial effects of metformin on biochemistry liver profile and metabolic parameters, but not on histological features.

The first study was conducted by Schwimmer et al. [63], who tested metformin (1 g/day) in ten insulin-resistant children with biopsy-proven steatohepatitis, for a period of 24 weeks. More recently, Naideau et al. [64] randomized fifty obese and insulin-resistant adolescents to receive lifestyle recommendations plus metformin (850 mg twice a day for 6 months) or placebo. In both studies the treatment with metformin resulted in serum aminotransferases, liver fat, and insulin sensitivity improvements as compared with untreated or placebo-treated group.

In another small observational trial conducted for 24 months in ten obese-overweight children with NAFLD [65], metformin (1.5 g/day) did not appear more effective than lifestyle intervention in ameliorating levels of aminotransferases and liver histology, but it significantly improved metabolic parameters and HOMA index. Similar results were obtained in a larger randomized multicenter placebo-controlled trial called the TONIC (Treatment of Nonalcoholic Liver Disease in Children) in which 57 children with NAFLD were treated with metformin (1 g/day) for 96 weeks. The study demonstrated that metformin is not superior to placebo in attaining a sustained reduction in ALT levels and significant improvements in histological features [66].

Although these clinical trials have sometimes shown controversial results, probably because they have generally been small, short term, and with often inconsistent outcomes, it is undoubted that metformin, by improving metabolic features of NAFLD, does show promise in the management of this liver disease. However, further larger randomized controlled trials of sufficient duration and using histological endpoints are needed to assess the effectiveness of this drug in modifying the natural history of NAFLD.

## 7. A Rational for the Use of Metformin in NAFLD

### 7.1. Effects on Metabolic Abnormalities and Cardiovascular Risk

As previously mentioned, NAFLD is now considered a hepatic manifestation of the metabolic syndrome. Patients with NAFLD frequently have many clinically significant co-morbidities, such as obesity, impaired glucose tolerance, type 2 diabetes, hypertension, and hyperlipidemia (high fasting serum triglyceride and LDL levels and low HDL values). The complex of these pathological conditions leads to an increased cardiovascular risk [23] and may contribute to the progression of hepatic damage. The therapeutical approach to NAFLD, therefore, aims at ameliorating these metabolic derangements, all linked to insulin resistance.

Treatment with an insulin-sensitizing agent, such as metformin, may correct several of these components of the metabolic syndrome. Moreover, in diabetic patients, metformin provides cardiovascular protection that cannot be attributed only to its antihyperglycemic effects. These additional cardioprotective effects may be related to the favorable actions of metformin on lipid metabolism, vascular smooth-muscle and cardiomyocyte intracellular calcium handling, endothelial function, hypercoagulation, and platelet hyperactivity [67].

Metformin therapy may result in a significant antihypertensive effects [68], which include both insulin-dependent and insulin-independent vasodilatory actions and probably also central antihypertensive effects [69]. Several authors have shown that metformin improves lipoprotein profiles with a decrease in low-density lipoprotein (LDL) cholesterol levels, triglycerides, and high-density lipoprotein (HDL) cholesterol levels [3]. Metformin has been reported to reduce markers of inflammation and to lessen hypercoagulation and increase fibrinolysis by decreasing levels of plasminogen activator inhibitor-1 and increasing tissue plasminogen activator activity. Metformin also improves functional and biochemical markers of endothelial reactivity as well as surrogate indexes of coronary atherosclerosis [67]. Another relevant mechanism is the reduction of circulating advanced glycated end products (AGEs), which are oxidative mediators of endothelial dysfunction. Moreover, metformin is able to stimulate intracellular AMPK and to activate the endothelial isoform of NOSs in human aortic endothelial cells [70].

### 7.2. Effects on Weight Loss

The reported prevalence of obesity in patients with NAFLD varies from 30 to 100% [8] and increases with increasing BMI. An analysis of liver histology suggests that the prevalence rates of steatosis and steatohepatitis are approximately 15% and 3%, respectively, in nonobese persons, while they increase to 65% and 20%, respectively, in patients with class I-II obesity (BMI: 30.0–39.9 kg/m^2^) and to 85% and 40%, respectively, in extremely obese patients (BMI: ≥40 kg/m^2^) [71].

These data support the rationale of using metformin, which help reducing body weight in obese patients with and without diabetes [72–75] and induces a significant reduction in total body fat and visceral fat [74]. Weight loss during metformin treatment has been attributed to decreased net caloric intake [76], probably through appetite suppression, an effect largely independent of gastrointestinal side effects of metformin [73]. Reduction in hyperinsulinemia related to reduced insulin resistance may have an additive effect on weight reduction in obese, insulin-resistant patients [77–79].

It has been reported that even a modest weight loss can produce improvements in markers for NAFLD, namely, ALT and imaging markers of liver fat [80, 81].

In the Diabetes Prevention Program, metformin use was associated with a small improvement in ALT levels over time. Weight loss appeared to be the dominant mediator of this effect, and the 4-year cumulative incidence for development of abnormal ALT values was lowest in patients who lost the most weight [82].

### 7.3. Effects on Glucose Disorders

The prevalence of T2DM in NAFLD varies from 10 and 75% [8], and this condition is the only independent variable associated with advanced-stage NAFLD [13]. Indeed, the great frequency of impaired fasting glucose and impaired glucose tolerance in NAFLD requires a therapeutic approach which delays and reduces the onset of diabetes. Metformin therapy contributes to protect pancreatic *β*-cell reserve and delay diabetes by lowering blood glucose and reducing peripheral insulin resistance.

In a large randomized placebo-controlled trial, the Diabetes Prevention Program (DPP) recently showed that improvement in insulin sensitivity, through either intensive lifestyle modification or metformin, reduces the risk of developing T2DM in high-risk individuals [83]. Data obtained in the same population demonstrated that metformin does not mask the development of diabetes but provides a curative effect on glucose derangement [78].

Recently, we compared the efficacy of a treatment with low-dose metformin and dietary measures alone in obese, nondiabetic patients with NAFLD in a 6-month, prospective, randomized study. After therapy, the proportion of patients with impaired fasting glucose declined from 35 to 5% (*P* = 0.04) in the metformin group, a proportion significantly higher in respect to the control group [35].

### 7.4. Effects on Polycystic Ovary Syndrome

PCOS represent a very common condition affecting 6-7% of reproductive aged women [84]. Insulin resistance has a pivotal role in ovulatory disfunction and androgen excess and represents a strong pathogenetic link with metabolic abnormalities associated with metabolic syndrome and NAFLD. Moreover, the prevalence of NAFLD in PCOS women ranges from 30 to 60% [85].

The pleiotropic action of metformin makes it a first-line medical therapy in PCOS women. Metformin decreases ovary production of total and free testosterone levels and improves follicular growth with both an indirect action, through the reduction of hyperinsulinemia, and a direct action on ovarian tissue, through the increase in AMPK and a reduction in CYP17 activity [84]. Clinically, metformin therapy improves hirsutism and normalizes menstrual cycles and induces ovulation in PCOS patients [67]. In PCOS, this approach with metformin introduced a pharmaceutical option targeting various aspects of this syndrome, which were previously neglected but that may contribute to adverse cardiometabolic outcomes.

### 7.5. Effects on Cancer Risk

T2DM and obesity are associated with an increased risk of a variety of cancers [86–91], while weight control is associated with a decreased cancer risk [92]. Recent data have elucidated some molecular mechanisms by which insulin resistance is involved in cancer [93]. Moreover, metabolic syndrome is associated with a worsen cancer outcome [94].

Hepatocellular carcinoma (HCC) is a complication of NAFLD-associated cirrhosis, and the majority of “cryptogenic” HCC in the United States is attributed to NAFLD [94]. Both T2DM and metabolic syndrome are also associated with HCC. However, it is unclear whether NAFLD predisposes patients to HCC in the absence of cirrhosis. Studies supported evidence that HCC may develop in NAFLD unaccompanied by cirrhosis [95].

These observations have several implications in NAFLD prevention and treatment: first because early treatment of NAFLD plays a role in primary prevention of HCC and second because metformin, itself may have an antitumor effect both in vitro and in vivo [91, 96]. In fact, T2DM patients, who are prescribed metformin, have a lower risk of cancer compared to patients that are not treated with metformin [97, 98].

The upstream regulator of AMPK is a protein kinase known as LKB1, a well-recognized tumor suppressor. Activation of AMPK by metformin and exercise requires LKB1, and this would also explain why exercise is beneficial in the primary and secondary prevention of certain cancers [97].

Metformin is known to activate AMPK and to inhibit cyclin D1 expression and proliferation of cultured cancer cells. However, the mechanisms of action by which metformin mediates cell cycle arrest are not completely understood [99].

In a retrospective case-control study performed in 465 HCC patients, it has been found that T2DM is an independent risk factor for HCC and pre-exists in the majority of HCC patients. Moreover, in patients with T2DM, there was a direct association of HCC with insulin and sulphonylureas treatment and an inverse relationship with metformin therapy [100]. In addition, the predictive value of hyperinsulinemia in total cancer mortality [101] and fatal liver tumor incidence [102] has been demonstrated in nondiabetic subjects by two recent prospective studies.

## 8. Monitoring the Efficacy of Metformin Therapy in NAFLD

As we previously mentioned, most studies with metformin show an improvement in liver aminotransferases and liver histology. However, it is still unclear what is the best way to monitor the response to therapy in NAFLD patients. In fact, despite an increase in transaminases is common in NAFLD, liver enzymes may be normal in up to 78% of patients and, thus, are insensitive not only for NAFLD diagnosis but also for disease monitoring. On the other hand, although liver biopsy is still considered the gold standard for distinguishing between the broad range of chronic liver diseases [19], it has several limitation, including cost, the potential risk of bleeding, and the absence of consensus regarding the histopathological criteria that firmly define NASH and differentiate between NAFLD entities [8, 103]. Accordingly, a repeated liver biopsy after a short term therapeutic trial is considered too invasive and impracticable to be applied in clinical practice, especially when considering the high prevalence of the disease [5].

Liver ultrasonography, the most practicable method for NAFLD detection, is not yet validated to monitor response to treatment. Thus, new sensitive and noninvasive markers of response are needed. As already mentioned, several markers have been proposed, such as fibrosis score and cytokines, but remain yet to be validated. Therefore, metabolic parameters still represent viable indexes of response to therapy.

## 9. Emerging Concepts and Candidate Future Therapies in NAFLD

Since the etiology and pathogenesis of NAFLD are not entirely clarified, new therapies await future developments in our understanding of key pathogenetic mechanisms of NAFLD. Ongoing research is exploring novel approaches that look promising in preclinical models.

MicroRNAs (miRNAs) have been recently studied in a rat model of NAFLD [104] and in cultured human hepatocytes [105] and seem to have an important role in hepatic energy metabolism and in the pathophysiological process of NAFLD. Several miRNAs are dysregulated in NAFLD, and this contributes to dysregulation of genes involved in hepatocyte proliferation, apoptosis, inflammation, and glucose and lipid metabolism [106]. The development of an effective and safe approach for correcting miRNA dysregulation is a new challenge for NAFLD therapy.

Novel chemically engineered oligonucleotides, termed “antagomirs,” have been shown to be efficient and specific silencers of endogenous miRNAs [107]. Particularly, both in monkeys [108] and in mice [107], antagomir against miRNA-122 resulted in a reduction of hepatic steatosis. A phase I study in humans is ongoing [106].

The regulation of AMPK activity is another emerging molecular target for the treatment of NAFLD. Pharmacological AMPK activators are being developed for the treatment of multiple metabolic disorders including NAFLD. Beside glucose homeostasis, AMPK also regulates hepatic lipid metabolism [106]. AMPK activation by AICAR or alpha-lipoic acid has been shown to decrease liver fat content in lean and obese rodents [109, 110]. These preclinical data need confirmation in human trials.

Decreased levels of glucagon-like peptide 1 (GLP-1) are common in obese patients [111], and reduced incretin action has been demonstrated in nondiabetic, nonobese patients with NASH, thus, prompting evaluation of GLP-1 analogs in fatty liver [106]. In obese mice, exendin, a GLP-1 agonist, may ameliorate insulin resistance and decrease histologic steatosis [112]. Only one case report illustrates a similar effect in humans [113]. Appropriate trials are, therefore, needed to asses the potential use of GLP-1 agonists in NAFLD [111].

In conclusion results of preclinical studies on emerging therapy of NAFLD are encouraging, but further work is needed to evaluate the efficacy and safety of these new agents in humans.

## 10. Conclusive Remarks

**Figure 2:**
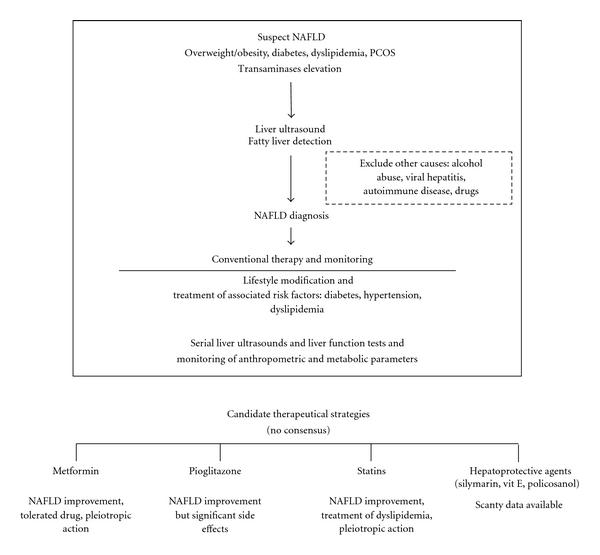
Schematic representation of NAFDL diagnosis and management. No consensus is available for the use of insulin sensitizers (metformin and pioglitazone), although studies have generally shown beneficial effects. The US Food and Drug Administration (FDA) has recently released a note to inform that the use of pioglitazone for more than one year may be associated with an increased risk of bladder cancer. In France the use of pioglitazone has been suspended while in Germany it is recommended not to start pioglitazone in new patients. Scanty data are available with regard to the efficacy of statins and hepatoprotective agents. Liver ultrasonography, the most practicable method for NAFLD detection is actually used to monitor response to treatment although not yet validated. Liver function tests may be useful despite a poor sensibility for NAFLD diagnosis and disease monitoring. Metabolic parameters still represent viable indexes of response to therapy.

No drug is currently available as specific treatment for NAFLD, and no drug can substitute for lifestyle modification. However, available evidences clearly show a pivotal role of metformin in improving metabolic alterations associated with NAFLD. Therefore, metformin, because of its metabolic effects and its safety profile, remains a promising drug in NAFLD therapy, especially in patients that meet the diagnostic criteria of metabolic syndrome [35]. A schematic representation of NAFLD diagnosis and management is shown in Figure 2.

## Figures and Tables

**Table 1:** Summary of metformin trials in NAFLD/NASH patients.

References	Study design	Patients	Therapy	Outcomes
Marchesini et al. [52]	OL, SA	20 patients, (OB); NASH, elevated AMTs	Metformin 1.5 g/d; 4 months	↓ ALT ↓ IR ↓ liver volume
Nair et al. [53]	OL, SA	28 patients, (OW/OB/T2DM); NAFLD	Metformin 20 mg/kg/d; 12 months	↓ ALT and AST ↓ IR Histology improved
Uygun et al. [54]	OL, RAND	36 patients, (OW/OB); NASH, elevated AMTs	Metformin 1.7 g/d + diet versus diet; 6 months	↓ IR ↓ ALT and AST Histology not improved
Bugianesi et al. [55]	OL, RAND (MC)	110 patients, (OW/OB/T2DM); NAFLD, elevated AMTs	Metformin 2 g/d + diet versus vit E + diet versus die; 12 months	↓ AST and ALT Histology improved
Schwimmer et al. [63]	SA	10 patients (OB/NT2DM children); NASH, elevated AMTs	Metformin 1 g/d; 6 months	↓ AST and ALT ↓ Liver fat ↓ IR
Duseja et al. [56]	OL, NRAND	50 patients, (OW/OB); NAFLD, elevated AMTs	Metformin 1.5 g/d; 6 months versus diet	↓ ALT and AST ↓ IR
Loomba et al. [47]	OL, SA	28 patients, (OW/OB/T2DM); NASH, elevated AMTs	Metformin 2 g/d; 12 months	Histology improved ↓ ALT and AST ↓ IR
de Oliveira et al. [57]	OL, SA	20 patients, (OW/OB/T2DM); NASH, elevated AMTs	Metformin 1 g/d; 12 months	↓ ALT Histology improved ↓ IR
Idilman et al. [58]	OL, RAND	74 patients, (OW/OB/T2DM); NASH, elevated AMTs	Metformin 1.7 g/d; 12 months	↓ ALT Histology not improved ↓ IR
Nobili et al. [65]	OL	57 patients (OW/OB children); NASH/NAFLD	Metformin 1.5 g/d versus diet; 24 months	↓ ALT and AST ↓ IR Histology improved
Haukeland et al. [59]	PLAC, RAND	48 patients (OW/OB/T2DM); NAFLD, elevated AMTs	Metformin versus placebo; 6 months	↓ ALT and AST ↓ IR Histology not improved
Nadeau et al. [64]	RAND	50 patients (OB children); NAFLD/elevated AMTs	Metformin 1.7 g/d + diet versus diet; 6 months	↓ ALT and AST ↓ IR Ultrasound pattern improved
Garinis et al. [35]	OL, RAND	50 patients, (OW/OB); NAFLD, normal AMTs	Metformin 1 g/d + diet versus diet; 6 months	↓ ALT and AST, ↓ IR Ultrasound pattern improved ↑ Adiponectin

Abbreviations: ALT, alanine transaminase; AMTs, aminotransferases; AST, aspartate aminotransferase; IR, insulin resistance; NAFLD, nonalcoholic fatty liver disease; NASH, nonalcoholic steatohepatitis; NRAND, non randomized; NT2DM, non type 2 diabetes mellitus; OB, obese; OL, open label; OW, overweight; PLAC, placebo controlled; RAND, randomized; SA, single arm; T2DM, type 2 diabetes mellitus.

## References

[1] Festi D, Colecchia A, Sacco T, Bondi M, Roda E, Marchesini G (2004). Hepatic steatosis in obese patients: clinical aspects and prognostic significance. *Obesity Reviews*.

[2] Marchesini G, Bugianesi E, Forlani G (2003). Nonalcoholic fatty liver, steatohepatitis, and the metabolic syndrome. *Hepatology*.

[3] Loria P, Lonardo A, Bellentani S, Day CP, Marchesini G, Carulli N (2007). Non-alcoholic fatty liver disease (NAFLD) and cardiovascular disease: an open question. *Nutrition, Metabolism and Cardiovascular Diseases*.

[4] de Alwis NMW, Day CP (2008). Non-alcoholic fatty liver disease: the mist gradually clears. *Journal of Hepatology*.

[5] Socha P, Horvath A, Vajro P, Dziechciarz P, Dhawan A, Szajewska H (2009). Pharmacological interventions for nonalcoholic fatty liver disease in adults and in children: a systematic review. *Journal of Pediatric Gastroenterology and Nutrition*.

[6] Schwimmer JB, Khorram O, Chiu V, Schwimmer WB (2005). Abnormal aminotransferase activity in women with polycystic ovary syndrome. *Fertility and Sterility*.

[7] Setji TL, Holland ND, Sanders LL, Pereira KC, Diehl AM, Brown AJ (2006). Nonalcoholic steatohepatitis and nonalcoholic fatty liver disease in young women with polycystic ovary syndrome. *Journal of Clinical Endocrinology and Metabolism*.

[8] Angulo P (2002). Medical progress: nonalcoholic fatty liver disease. *The New England Journal of Medicine*.

[9] Day CP, James OFW (1998). Steatohepatitis: a tale of two ’Hits’?. *Gastroenterology*.

[10] Matsuzawa N, Takamura T, Kurita S (2007). Lipid-induced oxidative stress causes steatohepatitis in mice fed an atherogenic diet. *Hepatology*.

[11] Gentile CL, Pagliassotti MJ (2008). The role of fatty acids in the development and progression of nonalcoholic fatty liver disease. *Journal of Nutritional Biochemistry*.

[12] Stefan N, Häring H-U (2011). The metabolically benign and malignant fatty liver. *Diabetes*.

[13] Mofrad P, Contos MJ, Haque M (2003). Clinical and histologic spectrum of nonalcoholic fatty liver disease associated with normal ALT values. *Hepatology*.

[14] Stefan N, Kantartzis K, Machann J (2008). Identification and characterization of metabolically benign obesity in humans. *Archives of Internal Medicine*.

[15] Fabbrini E, Magkos F, Mohammed BS (2009). Intrahepatic fat, not visceral fat, is linked with metabolic complications of obesity. *Proceedings of the National Academy of Sciences of the United States of America*.

[16] Akbar DH, Kawther AH (2006). Non-alcoholic fatty liver disease and metabolic syndrome: what we know and what we don’t know. *Medical Science Monitor*.

[17] Browning JD, Szczepaniak LS, Dobbins R (2004). Prevalence of hepatic steatosis in an urban population in the United States: impact of ethnicity. *Hepatology*.

[18] Kotronen A, Westerbacka J, Bergholm R, Pietilainen KH, Yki-Jarvinen H (2007). Liver fat in the metabolic syndrome. *The Journal of Clinical Endocrinology & Metabolism*.

[19] Wieckowska A, McCullough AJ, Feldstein AE (2007). Noninvasive diagnosis and monitoring of nonalcoholic steatohepatitis: present and future. *Hepatology*.

[20] Hamaguchi M, Kojima T, Takeda N (2005). The metabolic syndrome as a predictor of nonalcoholic fatty liver disease. *Annals of Internal Medicine*.

[21] Tobari M, Hashimoto E, Yatsuji S, Torii N, Shiratori K (2009). Imaging of nonalcoholic steatohepatitis: advantages and pitfalls of ultrasonography and computed tomography. *Internal Medicine*.

[22] Dasarathy S, Dasarathy J, Khiyami A, Joseph R, Lopez R, McCullough AJ (2009). Validity of real time ultrasound in the diagnosis of hepatic steatosis: a prospective study. *Journal of Hepatology*.

[23] Vuppalanchi R, Chalasani N (2009). Nonalcoholic fatty liver disease and nonalcoholic steatohepatitis: selected practical issues in their evaluation and management. *Hepatology*.

[24] Yamauchi T, Kamon J, Minokoshi Y (2002). Adiponectin stimulates glucose utilization and fatty-acid oxidation by activating AMP-activated protein kinase. *Nature Medicine*.

[25] Tsochatzis EA, Papatheodoridis GV, Archimandritis AJ (2009). Adipokines in nonalcoholic steatohepatitis: from pathogenesis to implications in diagnosis and therapy. *Mediators of Inflammation*.

[26] Tiniakos DG, Vos MB, Brunt EM (2010). Nonalcoholic fatty liver disease: pathology and pathogenesis. *Annual Review of Pathology*.

[27] Yoneda M, Fujita K, Inamori M (2007). Transient elastography in patients with non-alcoholic fatty liver disease (NAFLD). *Gut*.

[28] Angulo P, Hui JM, Marchesini G (2007). The NAFLD fibrosis score: a noninvasive system that identifies liver fibrosis in patients with NAFLD. *Hepatology*.

[29] Ratziu V, Giral P, Charlotte F (2000). Liver fibrosis in overweight patients. *Gastroenterology*.

[30] Dixon JB, Bhathal PS, O’Brien PE (2001). Nonalcoholic fatty liver disease: predictors of nonalcoholic steatohepatitis and liver fibrosis in the severely obese. *Gastroenterology*.

[31] Huang MA, Greenson JK, Chao C (2005). One-year intense nutritional counseling results in histological improvement in patients with nonalcoholic steatohepatitis: a pilot study. *American Journal of Gastroenterology*.

[32] Palmer M, Schaffner F (1990). Effect of weight reduction on hepatic abnormalities in overweight patients. *Gastroenterology*.

[33] Wang RT, Koretz RL, Yee HF (2003). Is weight reduction an effective therapy for nonalcoholic fatty liver? A systematic review. *American Journal of Medicine*.

[34] Kadayifci A, Merriman RB, Bass NM (2007). Medical treatment of non-alcoholic steatohepatitis. *Clinics in Liver Disease*.

[35] Garinis GA, Fruci B, Mazza A (2010). Metformin versus dietary treatment in nonalcoholic hepatic steatosis: a randomized study. *International Journal of Obesity*.

[36] Marlatt GA, Larimer ME, Mail PD (2003). Journeys of the circle: a culturally congruent life skills intervention for adolescent Indian drinking. *Alcoholism*.

[37] Tiikkainen M, Häkkinen AM, Korsheninnikova E, Nyman T, Mäkimattila S, Yki-Järvinen H (2004). Effects of rosiglitazone and metformin on liver fat content, hepatic insulin resistance, insulin clearance, and gene expression in adipose tissue in patients with type 2 diabetes. *Diabetes*.

[38] Ratziu V, Giral P, Jacqueminet S (2008). Rosiglitazone for nonalcoholic steatohepatitis: one-year results of the randomized placebo-controlled Fatty Liver Improvement with Rosiglitazone Therapy (FLIRT) Trial. *Gastroenterology*.

[39] Sanyal AJ, Mofrad PS, Contos MJ (2004). A pilot study of vitamin E versus vitamin E and pioglitazone for the treatment of nonalcoholic steatohepatitis. *Clinical Gastroenterology and Hepatology*.

[40] Belfort R, Harrison SA, Brown K (2006). A placebo-controlled trial of pioglitazone in subjects with nonalcoholic steatohepatitis. *The New England Journal of Medicine*.

[41] Aithal GP, Thomas JA, Kaye PV (2008). Randomized, placebo-controlled trial of pioglitazone in nondiabetic subjects with nonalcoholic steatohepatitis. *Gastroenterology*.

[42] Promrat K, Kleiner DE, Niemeier HM (2010). Randomized controlled trial testing the effects of weight loss on nonalcoholic steatohepatitis. *Hepatology*.

[43] Sanyal AJ, Chalasani N, Kowdley KV (2010). Pioglitazone, vitamin E, or placebo for nonalcoholic steatohepatitis. *The New England Journal of Medicine*.

[44] Chaffer CL, Thomas DM, Thompson EW, Williams ED (2006). PPAR*γ*-independent induction of growth arrest and apoptosis in prostate and bladder carcinoma. *BMC Cancer*.

[45] He L, Sabet A, Djedjos S (2009). Metformin and insulin suppress hepatic gluconeogenesis through phosphorylation of CREB binding protein. *Cell*.

[46] Stumvoll M, Nurjhan N, Perriello G, Dailey G, Gerich JE (1995). Metabolic effects of metformin in non-insulin-dependent diabetes mellitus. *The New England Journal of Medicine*.

[47] Loomba R, Lutchman G, Kleiner DE (2009). Clinical trial: pilot study of metformin for the treatment of non-alcoholic steatohepatitis. *Alimentary Pharmacology and Therapeutics*.

[48] Kohjima M, Higuchi N, Kato M (2008). SREBP-1c, regulated by the insulin and AMPK signaling pathways, plays a role in nonalcoholic fatty liver disease. *International Journal of Molecular Medicine*.

[49] Huypens P, Quartier E, Pipeleers D, Van De Casteele M (2005). Metformin reduces adiponectin protein expression and release in 3T3-L1 adipocytes involving activation of AMP activated protein kinase. *European Journal of Pharmacology*.

[50] Lin HZ, Yang SQ, Chuckaree C, Kuhajda F, Ronnet G, Diehl AM (2000). Metformin reverses fatty liver disease in obese, leptin-deficient mice. *Nature Medicine*.

[51] Zhang S, Liu X, Brickman WJ (2009). Association of plasma leptin concentrations with adiposity measurements in rural Chinese adolescents. *Journal of Clinical Endocrinology and Metabolism*.

[52] Marchesini G, Brizi M, Bianchi G, Tomassetti S, Zoli M, Melchionda N (2001). Metformin in non-alcoholic steatohepatitis. *Lancet*.

[53] Nair S, Diehl AM, Wiseman M, Farr GH, Perrillo RP (2004). Metformin in the treatment of non-alcoholic steatohepatitis: a pilot open label trial. *Alimentary Pharmacology and Therapeutics*.

[54] Uygun A, Kadayifci A, Isik AT (2004). Metformin in the treatment of patients with non-alcoholic steatohepatitis. *Alimentary Pharmacology and Therapeutics*.

[55] Bugianesi E, Gentilcore E, Manini R (2005). A randomized controlled trial of metformin versus vitamin E or prescriptive diet in nonalcoholic fatty liver disease. *American Journal of Gastroenterology*.

[56] Duseja A, Das A, Dhiman RK (2007). Metformin is effective in achieving biochemical response in patients with nonalcoholic fatty liver disease (NAFLD) not responding to lifestyle interventions. *Annals of Hepatology*.

[57] de Oliveira CPMS, Stefano JT, De Siqueira ERF (2008). Combination of N-acetylcysteine and metformin improves histological steatosis and fibrosis in patients with non-alcoholic steatohepatitis. *Hepatology Research*.

[58] Idilman R, Mizrak D, Corapcioglu D (2008). Clinical trial: insulin-sensitizing agents may reduce consequences of insulin resistance in individuals with non-alcoholic steatohepatitis. *Alimentary Pharmacology and Therapeutics*.

[59] Haukeland JW, Konopski Z, Eggesbø HB (2009). Metformin in patients with non-alcoholic fatty liver disease: a randomized, controlled trial. *Scandinavian Journal of Gastroenterology*.

[60] Janiec DJ, Jacobson ER, Freeth A, Spaulding L, Blaszyk H (2005). Histologic variation of grade and stage of non-alcoholic fatty liver disease in liver biopsies. *Obesity Surgery*.

[61] Omer Z, Cetinkalp S, Akyildiz M (2010). Efficacy of insulin-sensitizing agents in nonalcoholic fatty liver disease. *European Journal of Gastroenterology and Hepatology*.

[62] Nar A, Gedik O (2009). The effect of metformin on leptin in obese patients with type 2 diabetes mellitus and nonalcoholic fatty liver disease. *Acta Diabetologica*.

[63] Schwimmer JB, Behling C, Newbury R (2005). Histopathology of pediatric nonalcoholic fatty liver disease. *Hepatology*.

[64] Nadeau KJ, Ehlers LB, Zeitler PS, Love-Osborne K (2009). Treatment of non-alcoholic fatty liver disease with metformin versus lifestyle intervention in insulin-resistant adolescents. *Pediatric Diabetes*.

[65] Nobili V, Marcellini M, Devito R (2006). NAFLD in children: a prospective clinical-pathological study and effect of lifestyle advice. *Hepatology*.

[66] Lavine JE, Schwimmer JB, Molleston JP (2010). Treatment of nonalcoholic fatty liver disease in children: TONIC trial design. *Contemporary Clinical Trials*.

[67] Kirpichnikov D, McFarlane SI, Sowers JR (2002). Metformin: an update. *Annals of Internal Medicine*.

[68] Landin K, Tengborn L, Smith U (1991). Treating insulin resistance inhypertension with metformin reduces both blood pressure and metabolic risk factors. *Journal of Internal Medicine*.

[69] Petersen JS, DiBona GF (1996). Acute sympathoinhibitory actions of metformin in spontaneously hypertensive rats. *Hypertension*.

[70] Morrow VA, Foufelle F, Connell JMC, Petrie JR, Gould GW, Salt IP (2003). Direct activation of AMP-activated protein kinase stimulates nitric-oxide synthesis in human aortic endothelial cells. *Journal of Biological Chemistry*.

[71] Fabbrini E, Sullivan S, Klein S (2010). Obesity and nonalcoholic fatty liver disease: biochemical, metabolic, and clinical implications. *Hepatology*.

[72] Johansen K (1999). Efficacy of metformin in the treatment of NIDDM: meta-analysis. *Diabetes Care*.

[73] Haupt E, Knick B, Koschinsky T, Liebermeister H, Schneider J, Hirche H (1991). Oral antidiabetic combination therapy with sulphonylureas and metformin. *Diabete et Metabolisme*.

[74] Pasquali R, Gambineri A, Biscotti D (2000). Effect of long-term treatment with metformin added to hypocaloric diet on body composition, fat distribution, and androgen and insulin levels in abdominally obese women with and without the polycystic ovary syndrome. *Journal of Clinical Endocrinology and Metabolism*.

[75] Glueck CJ, Wang P, Fontaine R, Tracy T, Sieve-Smith L (1999). Metformin-induced resumption of normal menses in 39 of 43 (91%) previously amenorrheic women with the polycystic ovary syndrome. *Metabolism: Clinical and Experimental*.

[76] Yki-Järvinen H, Nikkilä K, Mäkimattila S (1999). Metformin prevents weight gain by reducing dietary intake during insulin therapy in patients with type 2 diabetes mellitus. *Drugs*.

[77] Sowers JR (1998). Obesity and cardiovascular disease. *Clinical Chemistry*.

[78] (2003). Effects of withdrawal from metformin on the development of diabetes in the diabetes prevention program. *Diabetes Care*.

[79] McFarlane SI, Banerji M, Sowers JR (2001). Insulin resistance and cardiovascular disease. *Journal of Clinical Endocrinology and Metabolism*.

[80] Hickman IJ, Jonsson JR, Prins JB (2004). Modest weight loss and physical activity in overweight patients with chronic liver disease results in sustained improvements in alanine aminotransferase, fasting insulin, and quality of life. *Gut*.

[81] Larson-Meyer DE, Newcomer BR, Heilbronn LK (2008). Effect of 6-month calorie restriction and exercise on serum and liver lipids and markers of liver function. *Obesity*.

[82] Krakoff J, Clark JM, Crandall JP (2010). Effects of metformin and weight loss on serum alanine aminotransferase activity in the diabetes prevention program. *Obesity*.

[83] Knowler WC, Barrett-Connor E, Fowler SE (2002). Reduction in the incidence of type 2 diabetes with lifestyle intervention or metformin. *The New England Journal of Medicine*.

[84] Diamanti-Kandarakis E (2008). Polycystic ovarian syndrome: pathophysiology, molecular aspects and clinical implications. *Expert Reviews in Molecular Medicine*.

[85] Gutierrez-Grobe Y, Ponciano-Rodríguez G, Ramos MH, Uribe M, Méndez-Sánchez N (2010). Prevalence of non alcoholic fatty liver disease in premenopausal, posmenopausal and polycystic ovary syndrome women. The role of estrogens. *Annals of Hepatology*.

[86] Chodick G, Heymann AD, Rosenmann L (2010). Diabetes and risk of incident cancer: a large population-based cohort study in Israel. *Cancer Causes and Control*.

[87] Vigneri P, Frasca F, Sciacca L, Frittitta L, Vigneri R (2006). Obesity and cancer. *Nutrition, Metabolism and Cardiovascular Diseases*.

[88] Strickler HD, Wylie-Rosett J, Rohan T (2001). The relation of type 2 diabetes and cancer. *Diabetes Technology and Therapeutics*.

[89] Coughlin SS, Calle EE, Teras LR, Petrelli J, Thun MJ (2004). Diabetes mellitus as a predictor of cancer mortality in a large cohort of US adults. *American Journal of Epidemiology*.

[90] Renehan AG, Tyson M, Egger M, Heller RF, Zwahlen M (2008). Body-mass index and incidence of cancer: a systematic review and meta-analysis of prospective observational studies. *The Lancet*.

[91] Cantrell LA, Zhou C, Mendivil A, Malloy KM, Gehrig PA, Bae-Jump VL (2010). Metformin is a potent inhibitor of endometrial cancer cell proliferation-implications for a novel treatment strategy. *Gynecologic Oncology*.

[92] Vainio H, Kaaks R, Bianchini F (2002). Weight control and physical activity in cancer prevention: international evaluation of the evidence. *European Journal of Cancer Prevention*.

[93] Belfiore A, Frasca F (2008). IGF and insulin receptor signaling in breast cancer. *Journal of Mammary Gland Biology and Neoplasia*.

[94] Siegel AB, Zhu AX (2009). Metabolic syndrome and hepatocellular carcinoma: two growing epidemics with a potential link. *Cancer*.

[95] Guzman G, Brunt EM, Petrovic LM, Chejfec G, Layden TJ, Cotler SJ (2008). Does nonalcoholic fatty liver disease predispose patients to hepatocellular carcinoma in the absence of cirrhosis?. *Archives of Pathology and Laboratory Medicine*.

[96] Zakikhani M, Dowling R, Fantus IG, Sonenberg N, Pollak M (2006). Metformin is an AMP kinase-dependent growth inhibitor for breast cancer cells. *Cancer Research*.

[97] Evans JMM, Donnelly LA, Emslie-Smith AM, Alessi DR, Morris AD (2005). Metformin and reduced risk of cancer in diabetic patients. *British Medical Journal*.

[98] Bowker SL, Majumdar SR, Veugelers P, Johnson JA (2006). Increased cancer-related mortality for patients with type 2 diabetes who use sulfonylureas or insulin. *Diabetes Care*.

[99] Zhuang Y, Miskimins WK (2008). Cell cycle arrest in Metformin treated breast cancer cells involves activation of AMPK, downregulation of cyclin D1, and requires p27Kip1 or p21Cip1. *Journal of Molecular Signaling*.

[100] Donadon V, Balbi M, Ghersetti M (2009). Antidiabetic therapy and increased risk of hepatocellular carcinoma in chronic liver disease. *World Journal of Gastroenterology*.

[101] Verlato G, Zoppini G, Bonora E, Muggeo M (2003). Mortality from site-specific malignancies in type 2 diabetic patients from Verona. *Diabetes Care*.

[102] Balkau B, Kahn HS, Courbon D, Eschwège E, Ducimetière P (2001). Hyperinsulinemia predicts fatal liver cancer but is inversely associated with fatal cancer at some other sites: the Paris Prospective Study. *Diabetes Care*.

[103] Gaidos JKJ, Hillner BE, Sanyal AJ (2008). A decision analysis study of the value of a liver biopsy in nonalcoholic steatohepatitis. *Liver International*.

[104] Jin X, Ye YF, Chen SH, Yu CH, Liu J, Li YM (2009). MicroRNA expression pattern in different stages of nonalcoholic fatty liver disease. *Digestive and Liver Disease*.

[105] Cheung O, Puri P, Eicken C (2008). Nonalcoholic steatohepatitis is associated with altered hepatic MicroRNA expression. *Hepatology*.

[106] Musso G, Gambino R, Cassader M (2010). Emerging molecular targets for the treatment of nonalcoholic fatty liver disease. *Annual Review of Medicine*.

[107] Krützfeldt J, Rajewsky N, Braich R (2005). Silencing of microRNAs in vivo with ‘antagomirs’. *Nature*.

[108] Elmén J, Lindow M, Schütz S (2008). LNA-mediated microRNA silencing in non-human primates. *Nature*.

[109] Bergeron R, Previs SF, Cline GW (2001). Effect of 5-aminoimidazole-4-carboxamide-1-*β*-d-ribofuranoside infusion on in vivo glucose and lipid metabolism in lean and obese zucker rats. *Diabetes*.

[110] Park KG, Min AK, Koh EH (2008). Alpha-lipoic acid decreases hepatic lipogenesis through adenosine monophosphate-activated protein kinase (AMPK)-dependent and AMPK-independent pathways. *Hepatology*.

[111] Lewis JR, Mohanty SR (2010). Nonalcoholic fatty liver disease: a review and update. *Digestive Diseases and Sciences*.

[112] Ding X, Saxena NK, Lin S, Gupta N, Anania FA (2006). Exendin-4, a glucagon-like protein-1 (GLP-1) receptor agonist, reverses hepatic steatosis in ob/ob mice. *Hepatology*.

[113] Tushuizen ME, Bunck MC, Pouwels PJ, van Waesberghe JHT, Diamant M, Heine RJ (2006). Incretin mimetics as a novel therapeutic option for hepatic steatosis. *Liver International*.

